# Cyclophilin D: Guardian or Executioner for Tumor Cells?

**DOI:** 10.3389/fonc.2022.939588

**Published:** 2022-07-04

**Authors:** Ling Zhang, Yi Liu, Rou Zhou, Baoyu He, Wenjun Wang, Bin Zhang

**Affiliations:** ^1^ School of Nursing, Jining Medical University, Jining, China; ^2^ School of Public Health, North China University of Science and Technology, Tangshan, China; ^3^ Department of Laboratory Medicine, Affiliated Hospital of Jining Medical University, Jining Medical University, Jining, China; ^4^ Institute of Forensic Medicine and Laboratory Medicine, Jining Medical University, Jining, China

**Keywords:** cyclophilin D, mitochondrial permeability transition pore, tumor energy metabolism, tumor cell death, tumor metastasis and invasion, tumor resistance

## Abstract

Cyclophilin D (CypD) is a peptide-proline cis-trans isomerase (PPIase) distributed in the mitochondrial matrix. CypD regulates the opening of the mitochondrial permeability conversion pore (mPTP) and mitochondrial bioenergetics through PPIase activity or interaction with multiple binding partners in mitochondria. CypD initially attracted attention due to its regulation of mPTP overopening-mediated cell death. However, recent studies on the effects of CypD on tumors have shown conflicting results. Although CypD has been proven to promote the aerobic glycolysis in tumor cells, its regulation of malignant characteristics such as the survival, invasion and drug resistance of tumor cells remains controversial. Here, we elaborate the main biological functions of CypD and its relationships with tumor progression identified in recent years, focusing on the dual role of CypD in tumors.

## Introduction

Cyclophilin D (CypD) is a cyclophilin distributed in the mitochondrial matrix that acts as the gatekeeper of mitochondria (1, 2). Most previous studies considered that CypD plays a vital role in regulating cell apoptosis or necrosis by regulating mitochondrial permeability. Early studies on CypD were mostly limited to ischaemia-reperfusion injury, neurodegeneration, ageing, diabetes, etc (3–6). With the in-depth research on CypD function, the relationship between CypD and tumors has gradually become a research hotspot in recent years. It remains controversial whether CypD promotes or inhibits tumor progression. Many studies on the regulation by CypD of tumor cell survival, invasion and drug resistance have reported inconsistent conclusions. It has been reported that CypD can affect the malignant characteristics of tumor progression by regulating the bioenergetics and mitochondrial permeability of tumor cells. Here, we review the biological functions of CypD and its role in tumor progression. In particular, the question of whether CypD is the guardian of tumor progression or the executioner of tumor treatment is fully expounded upon this review.

## Characteristics of CypD

CypD is a peptide-proline cis-trans isomerase (PPIase) widely expressed in all mammalian tissues. CypD is encoded by the nuclear gene *Ppif* (located at 10q22-10q23), and its mRNA is 2,213 bases in length and encodes 207 amino acids (aa). After shearing, it is translocated to the mitochondrial matrix and transformed into a 178-aa mature peptide. CypD was originally named mitochondrial cyclophilin (CYP-M) and is now sometimes referred to as cyclophilin F or cyclophilin 3 (7). It should be noted, however, that CypD has been used in the past to refer to a 370-aa cytoplasmic cyclophilin protein encoded by the *Ppid* gene. Under electron microscopy, CypD shows a spindle or rod structure with a diameter of 0.5-1.0 μm. CypD isomers are rarely reported.

The maintenance of normal mitochondrial permeability is critical to mitochondrial function and is mainly controlled by the status of the mitochondrial permeability transition pore (mPTP) (8, 9). mPTP, also known as the mitochondrial giant channel, is a calcium-dependent nonselective highly conductive channel. As early as the 1990s, CypD attracted attention due to its regulatory role in mPTP. The binding partners of CypD are mostly located in the mitochondrial inner membrane (MIM), including adenine nucleotide transporter (ANT), phosphate carrier (PiC), and oligomycin-sensitive binding protein (OSCP, a subunit of F_1_F_O_ ATP synthase) (9). CypD binding with its binding partners can induce a transient low level of mPTP opening, promoting ROS excretion out of mitochondria and maintaining mitochondrial calcium homeostasis. Upregulated expression or activity of CypD can provoke mPTP overopening and lead to the influx of a large number of substances with molecular weights less than 1.5 kDa into the mitochondrial matrix, resulting in mitochondrial membrane depolarization, oxidative phosphorylation (OXPHOS) uncoupling, ATP depletion, and the release of proapoptotic factors, subsequently inducing apoptosis or necrosis (10). Thus, abnormal activation of the CypD-mPTP axis is considered to be the executioner in various diseases, such as ischaemia/reperfusion injury, ageing and neurodegeneration. However, it has been reported that CypD overexpression inhibits ANT-mediated apoptosis, enhances cell resistance to harmful stimuli, and promotes cell survival (11). Therefore, for mitochondria and cells, the identity of CypD remains a mystery.

## The Major Biological Functions of CypD

### Regulation of Mitochondrial Permeability

**Figure 1:**
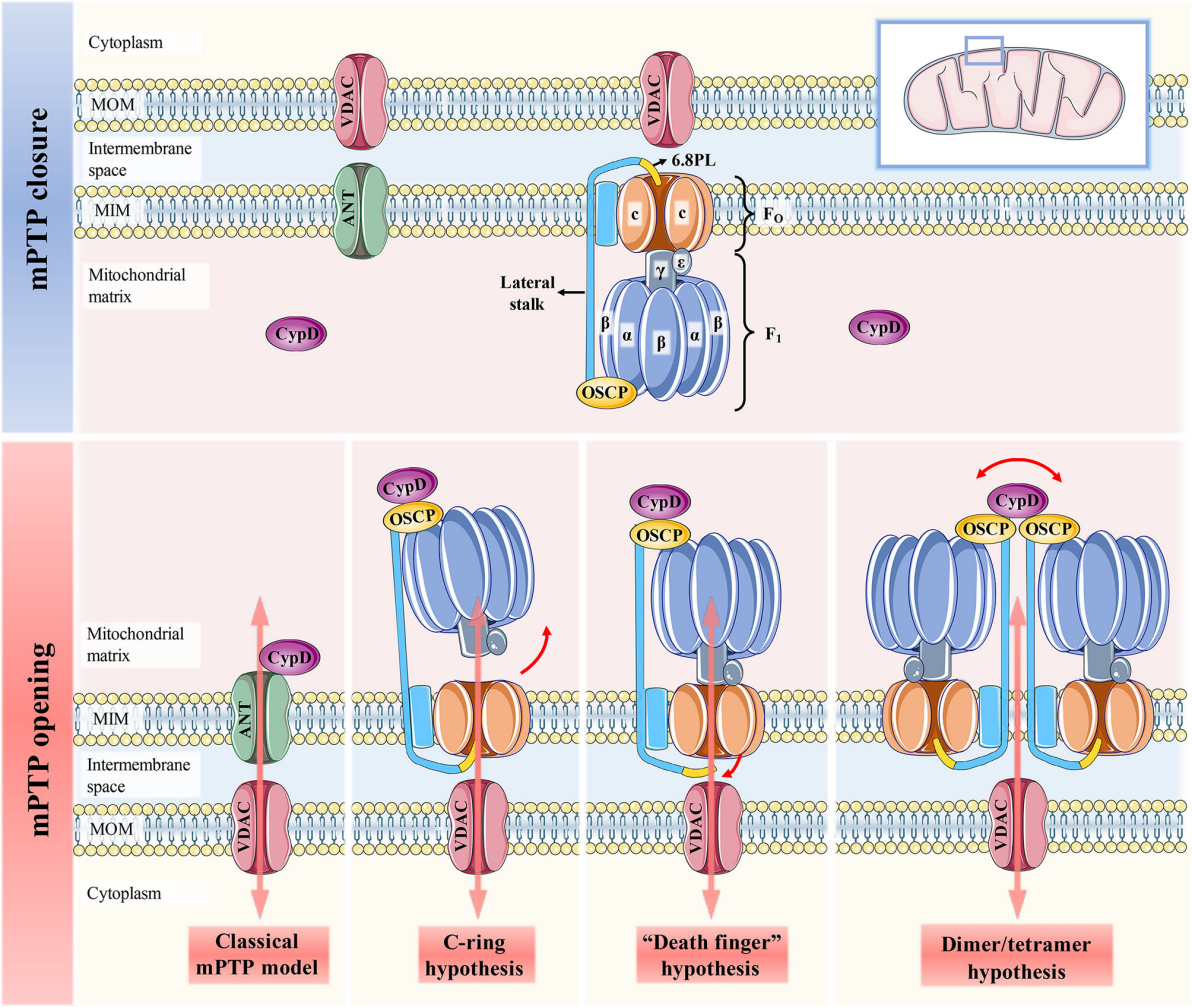
Schematic representation of the four mPTP models. When CypD is free in the mitochondrial matrix, mPTP is not activated and remains completely closed. The classical mPTP model holds that when CypD translocates from the matrix to the MIM and interacts with ANT, which can further induce the binding of ANT and VDAC to open the mPTP. The c-ring hypothesis suggests that conformational changes of F_1_ caused by CypD-OSCP association abolish the sealing effect of F_1_ on the c-ring, thereby promoting the central hole of the c-ring to form mPTP. Both the “dead finger” hypothesis and the dimer/tetramer hypothesis are caused by the conformation of the lateral stalk induced by the CypD-OSCP association, but the mPTP formation sites of the two are different. The former considers disassociating 6.8PL from c-ring to form mPTP. mPTP is formed by the lateral stalks of all F_1_F_O_ ATP synthases that make up the dimer/tetramers. The former suggests that mPTP is activated by the removal of 6.8PL from the c-ring, while the latter suggests that mPTP is formed by the surrounding lateral stalks of all F_1_F_O_ ATP synthases in the dimers/tetramers.

mPTP is regarded as a key effector of cell death, where many signals that regulate cell death converge. The physiological and pathological roles of mPTP have been well studied, but its molecular identity and necessary regulatory factors remain controversial. mPTP was initially thought to be mainly composed of voltage-dependent anion channels [VDAC, located in the mitochondrial outer membrane (MOM)], ANT and CypD and is regulated by hexokinase II (HK II), PiC, mitochondrial creatine kinase (mtCK) and other proteins (9, 12) (**Figure 1**). However, with further research, the credibility of the classical mPTP model has been questioned in a series of genetic knockout studies (13, 14). In the absence of ANT or VDAC, increased Ca^2+^ still activates mPTP, and does not prevent cell death caused by mPTP overopening. These results suggest that CypD may also associate with other binding partners to promote mPTP opening and influence mitochondrial permeability. It is worth to mention that CypD is the only genetically proven indispensable mPTP component (15).

It has been reported that mPTP is more likely to be located in the F_1_F_O_ ATP synthase (complex V of OXPHOS). At present, there are three hypotheses about the formation of mPTP by F_1_F_O_ ATP synthase, namely, the c-ring hypothesis, “death finger” hypothesis and dimer/tetramer hypothesis (16) (**Figure 1** **)**. The initiation signal of these three hypotheses is the mechanical combination of CypD and OSCP located in the crown of F_1_, but its downstream effect events are quite different (1): The c-ring hypothesis suggests that CypD-OSCP association induces the conformational changes in the F_1_ β subunit, decouples F_1_ and F_O_, and exposes the central hole of the c-ring to form mPTP (17) (2). The “death finger” hypothesis suggests that the mechanical force generated by CypD-OSCP association can be transmitted along the lateral stalk to the base of F_O_ and remove the sealing effect of the 6.8PL subunit on the c-ring (18, 19) (3). The dimer/tetramer hypothesis also derives from CypD-mediated lateral stalk conformational changes, which allow adjacent e and g subunits on the dimer/tetramer to form mPTP together (20). It has been reported that the inhibition of the β subunit on the c-ring can be relieved even without Ca^2+^ overload. Therefore, the pore formed in the c-ring hypothesis is not true mPTP, given that the formation of mPTP strictly requires Ca^2+^ for activation (21). In contrast, the status of mPTP in the other two hypotheses can be affected by Ca^2+^ concentration (22). The CypD-OSCP association not only induces conformational changes in F_1_F_O_ ATP synthase to form mPTP, but also enhances the affinity of Ca^2+^ to the binding site on the β subunit, and significantly downregulates the Ca^2+^ threshold that makes mPTP open (23). If either hypothesis is true, it means that mPTP is located only in the MIM. This is also recognized by most researchers, and they believe that mPTP is actually a large nonselective channel formed in MIM, which is apparently different from the traditional definition of mPTP (8, 10). However, the results of Carroll et al. cast doubt on the credibility of these three hypotheses (24). This is because the pore survives and can be induced to open by Ca^2+^ overload in mitochondria even after the removal of different subunits of the c-ring or lateral stalk. Thus, they concluded that any form or component of F_1_F_O_ ATP synthase was unlikely to form mPTP. The reason for this discrepancy may be that the purity, stability and functionality of F_1_F_O_ ATP synthase may differ to varying degrees from those *in vivo* when isolated from mitochondria and reconstructed into extracorporeal membrane systems for independent study (19). Regardless of the specific location of mPTP, the excessive activation of the CypD-mPTP axis always triggers the permeability of MIM and MOM sequentially and ultimately initiates the cascade of cell death signals.

Additionally, the cell fate regulation by CypD is controversial. It has been suggested that CypD is a signalling molecule that promotes cell survival. This is because the overexpression of CypD delays the occurrence of mitochondrial membrane potential (MMP) collapse in HEK293 and B50 cells induced by oxidative stress and astrosporin, preserves the integrity of the mitochondrial membrane and promotes cell survival. This difference may be caused by the fact that PPIase activity is required for the protective effect of CypD, while the CypD-mediated mPTP opening is independent of PPIase activity. Thus, CypD may maintain physiological mitochondrial permeability and reverse the fate of apoptosis by binding to its binding partners other than ANT or F_1_F_O_ ATP synthase. The specific identity and regulatory mechanisms of the binding partners involved in CypD-mediated protection need to be further explored.

### Regulation of Mitochondrial Energy Metabolism

It should be noted that F_1_F_O_ ATP synthase is also a key site for catalysing ATP production from ADP and inorganic phosphate (Pi). The conformation of the catalytic site on the β subunit can be altered when the protons generated by electron transport flows through the c-ring to drive ATP synthesis. The CypD-OSCP association not only induces the formation of mPTP, but also blocks the driving effect of the c-ring on F_1_ and interferes with ATP metabolism (8, 25, 26). To improve energy metabolism efficiency, F_1_F_O_ ATP synthase also combines with ANT and PiC to assemble a bioactive unit with a molecular weight of at least 700,000, called the ATP synthasome (27, 28). Compared with wild-type mice, the formation of the ATP synthasome was enhanced in the heart mitochondria of CypD^-/-^ mice, as well as in the brain and liver. In tissues with higher energy requirements (such as the heart and brain), F_1_F_O_ ATP synthase is more oligomerized, and CypD expression is lower (8). This is because CypD promotes the disassembly of the ATP synthasome and reduction to monomer or dimer forms, which is beneficial for mPTP opening. Therefore, no matter what the specific molecular nature of mPTP is, the regulatory role of CypD on mPTP opening cannot be overcome. The influences of the CypD-mPTP axis on mitochondrial permeability and energy metabolism modulate and promote each other, jointly determining cells fate.

**Figure 2:**
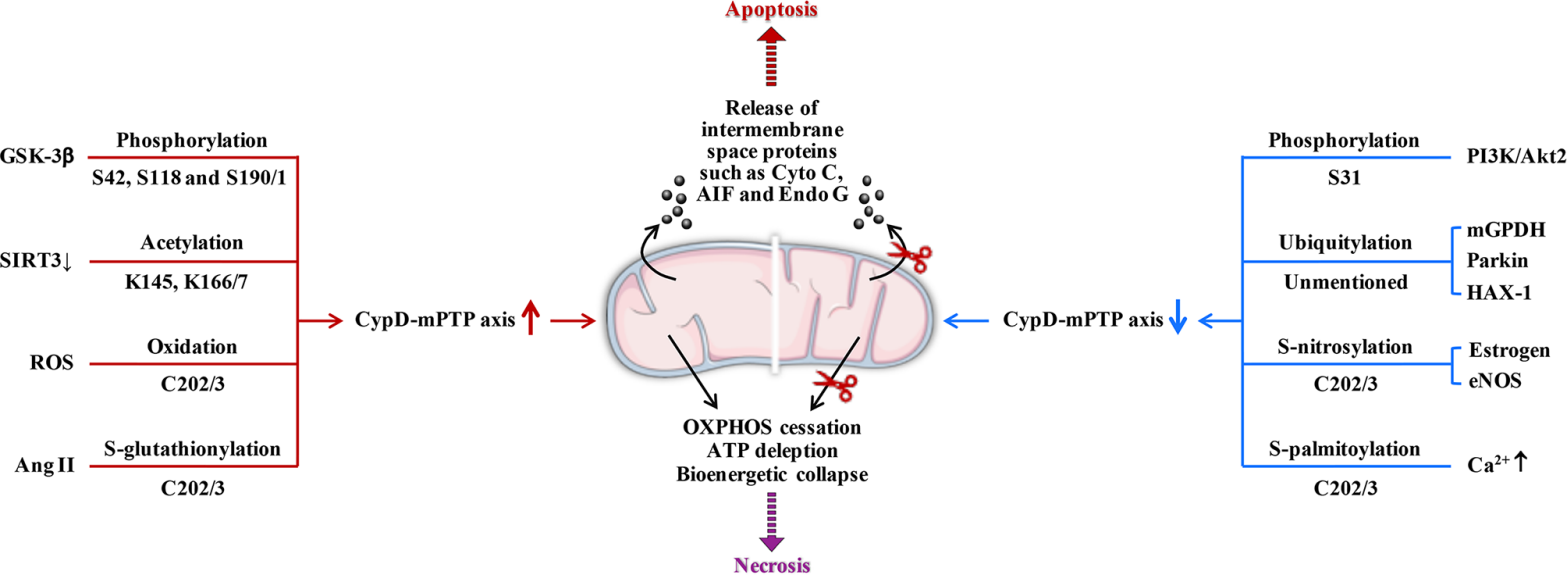
Posttranslational modifications of CypD. Overall, phosphorylation (S42 and S191), S-acetylation, oxidation, and S-glutathionylation (several sites) can upregulate CypD activity and sensitize mPTP to induce cell death. In contrast, phosphorylation (S31), ubiquitination, S-nitrosylation, S-palmitoylation, and S-glutathionylation (other sites) may downregulate CypD activity and provide cellular protection by desensitizing mPTP. S190/1, K166/7, and C202/3 represent homologous serine, lysine, and cysteine residues in the mouse/human form of CypD, respectively. GSK-3β, glycogen synthase kinase-3β; SIRT3, sirtuin3; ROS, reactive oxygen species; Ang II, angiotensin II; PI3K, phosphatidylinositol 3-hydroxy kinase; Akt2, Ser/Thr kinase; mGPDH, mitochondrial glycerol 3-phosphate dehydrogenase; HAX-1, haematopoietic-substrate-1 associated protein X-1; eNOS, endothelial nitric oxide synthase; AIF, apoptosis inducing factor; Endo G, endonuclease G; ↑, upregulation; ↓, downregulation.

**Figure 3:**
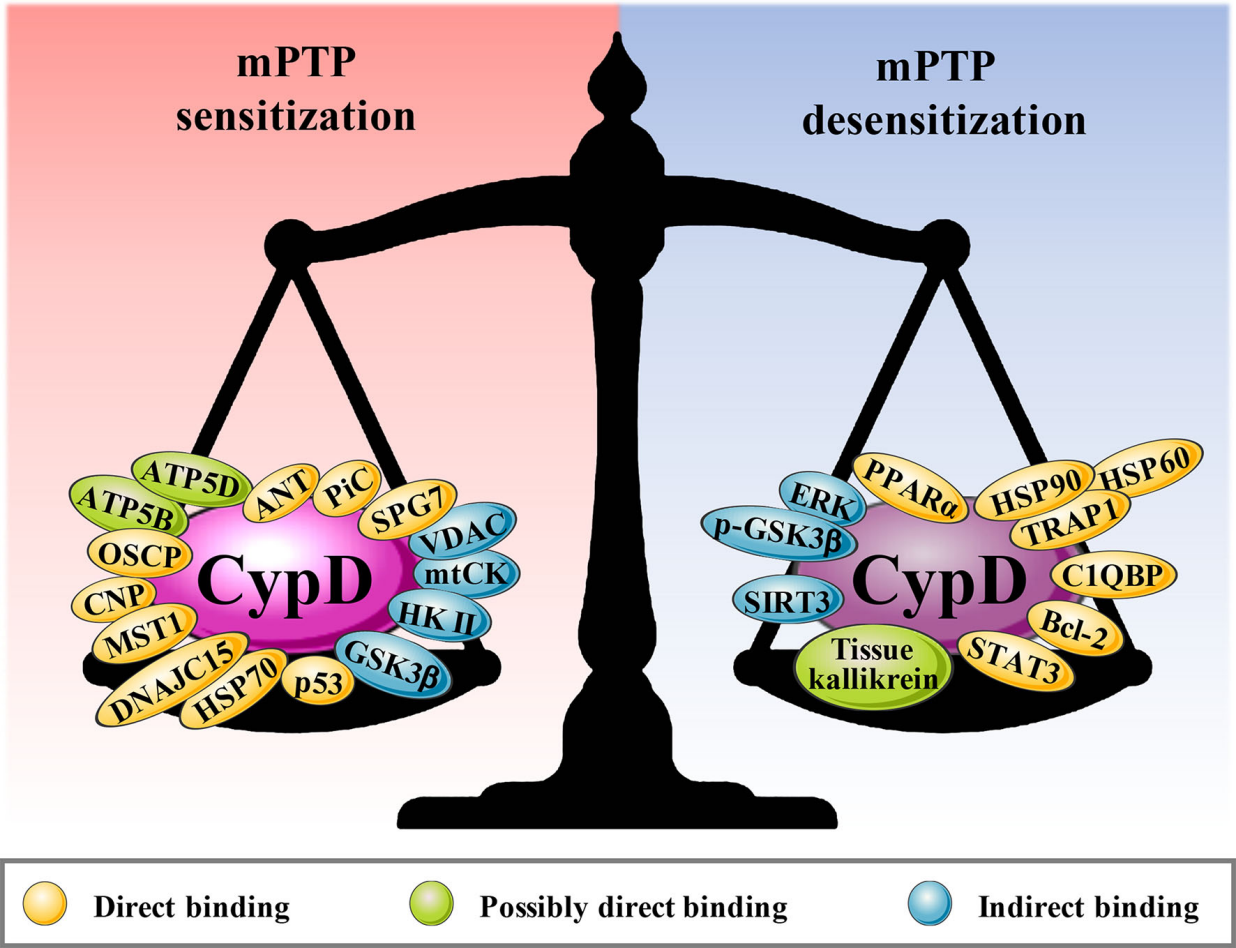
The binding partners of CypD. Red areas are the binding partners that sensitize mPTP, and blue areas are the binding partners that desensitize mPTP.

CypD activity is regulated by posttranslational modifications (PTMs) on multiple specific residues (29–36). The same PTM at different residues or different PTMs at the same residue may have different effects on CypD activity, translocation and its ability to regulate mPTP opening (**Figure 2**). Additionally, CypD can bind to multiple direct or indirect binding partners in the mitochondrial matrix to activate or inhibit the CypD-mPTP axis, as described in the review by Porter et al. (7) (**Figure 3**).

## Positive effect of CypD on Tumor Progression

### Promoting Tumorigenesis

The oncogene Ras was reported to enhance CypD expression through the Raf-1/MEK/ERK pathway (37). Upregulated expression of CypD could antagonize the inhibitory effect of the p53-p21 pathway on tumor cell growth and induce Ras-mediated tumorigenesis. The study also found that the oncogenic activity of CypD is p53 dependent. Inhibition or knockout of CypD can effectively prevent Ras-dependent lung cancer and Erbb2-mediated breast cancer formation.

### Maintaining Aerobic Glycolysis

Different from normal cells, tumor cells still have strong glycolysis activity and high acid metabolites even under the condition of adequate oxygen, which is known as the Warburg effect (38). The metabolic transition from OXPHOS to aerobic glycolysis reduces the dependence of tumor cells on oxygen availability and is beneficial to the survival and invasion of tumor cells in the hypoxic microenvironment. The enhancement of glycolytic activity in tumor cells is closely related to irreversible damage to OXPHOS. The interaction between CypD and different components of the ATP synthasome can interfere with the progression of OXPHOS in tumor cells to a certain extent but does not cause ATP depletion. The initiation of aerobic glycolysis in tumor cells requires ATP produced by OXPHOS as the substrate, which is different from traditional anaerobic glycolysis (39). Hexokinase (HK), as the first rate-limiting enzyme of glycolysis, consists of four subtypes. It is worth noting that HK II is widely expressed in embryonic tissues and invasive tumors but is rarely expressed in normal tissues and only sparingly expressed in insulin-sensitive tissues (fat, muscle, heart) (40, 41). HK II can translocate from the cytoplasm to the MOM and bind with VDAC, which is called mitochondrial HK II (mtHK II) (42). mtHK II limits the movement of the N-terminal spiral of VDAC and keeps VDAC open. This ensures that ATP produced within the mitochondrial matrix is preferentially and continuously transported to mtHK II for aerobic glycolysis (43).

It has been reported that CypD overexpression can promote the formation of mtHK II, while inhibition or knockdown of CypD inhibits the level of HK II binding to the MOM (42, 44). Upregulated sirtuin 3 (SIRT3) was confirmed to inhibit the acetylation degree and PPIase activity of CypD and lead to the separation of CypD from ANT in breast cancer cells treated with oroxylin A (45). Simultaneously, the deacetylation of CypD triggered the dissociation of mtHK II from the MOM and inhibited the aerobic glycolysis activity of breast cancer cells. Similarly, ganoderic acid D (GAD) inhibited mtHK II formation and energy reprogramming in colon cancer cells by inducing SIRT3-mediated CypD deacetylation (46). Inhibition of SIRT3 activity effectively reversed mitochondrial cytotoxicity and reduced aerobic glycolysis induced by the abovementioned antitumor drugs. Intriguingly, overexpression of mutant CypD did not significantly change the level of mtHK II impaired by oroxylin A. Moreover, glioma cells expressing PPIase-deficient CypD were not effective against Bax-induced apoptosis compared to cells overexpressing wild-type CypD (47). Therefore, it is reasonable to assume that CypD relies on its PPIase activity to stabilize the association of ANT-VDAC-mtHK II.

**Figure 4:**
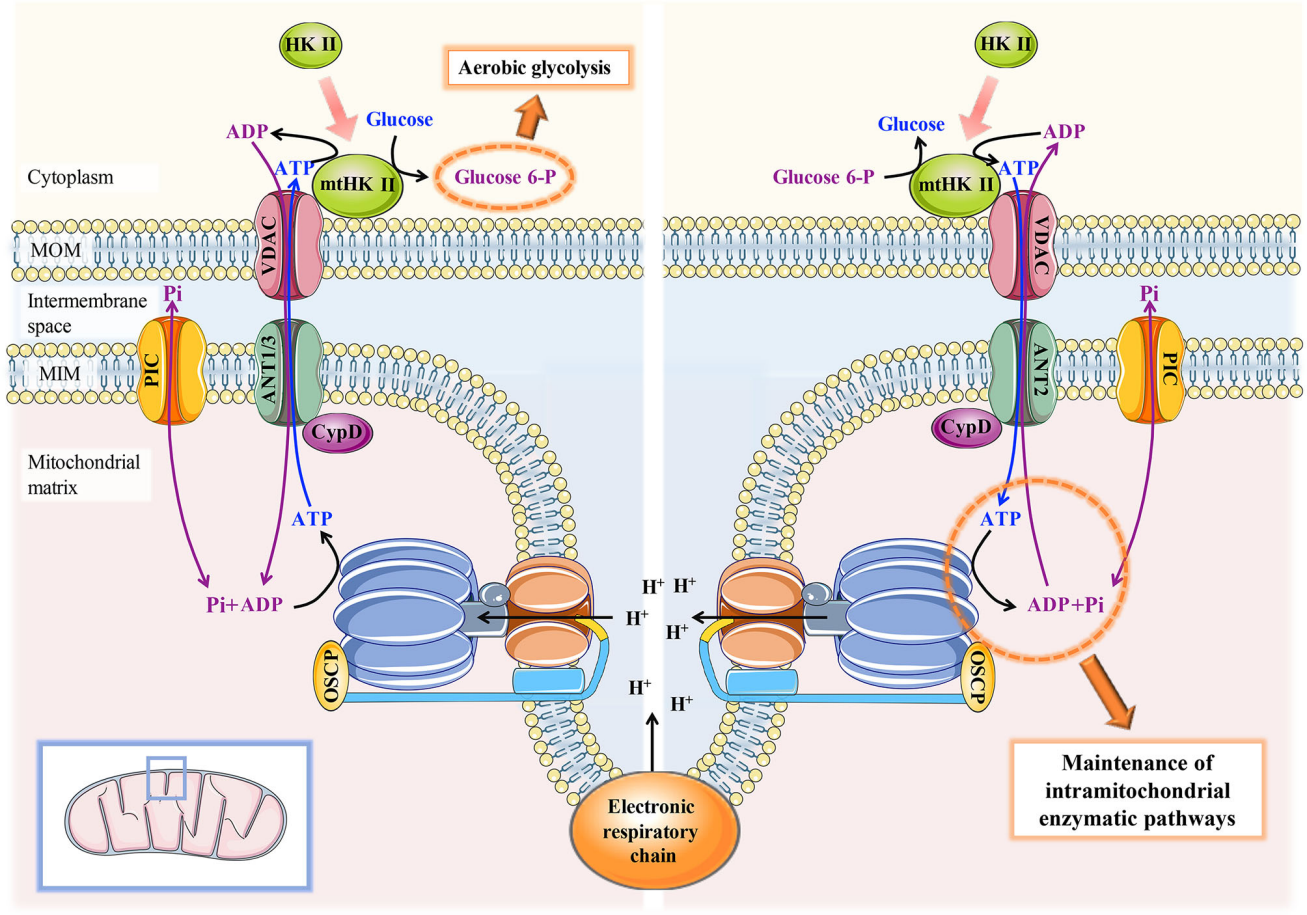
Schematic diagram of the mechanism of the CypD-ANT-VDAC-mtHK II association in the maintenance of aerobic glycolysis and mitochondrial function in tumor cells. Left: CypD-ANT1/3 association helps stabilize the binding of mtHKII to VDAC and thereby moderately activates mPTP, which ensures that ATP generated by OXPHOS can be preferentially and continuously transported to mtHK II for aerobic glycolysis. Right: conversely, mtHK II can reverse catalyse ATP production from G6P to maintain the essential intramitochondrial enzymatic pathways in tumor cells when the ATP concentration in the cytoplasm drops to a certain threshold. The maintenance of the latter process requires the CypD-ANT2-VDAC-mtHKII association.

An important question is why CypD-ANT binding is inconsistent in its dependence on PPIase activity in tumor and nontumor cells, which has been attributed to different ANT subtypes. There are four different subtypes of human ANT, including ANT1, ANT2, ANT3, and ANT4. Compared with the other three, ANT2 is widely expressed in rapidly growing cells, and only ANT2 can be highly induced in tumor cells (48, 49). The ANT2-VDAC-mtHK II association in tumor cells has been reported to play an important role in the maintenance of aerobic glycolysis and other carcinogenic effects (50, 51). Since ATP is continuously exported to the cytoplasm, it is not conducive to the maintenance of the essential intramitochondrial enzymatic pathways in tumor cells. Thus, mtHK II can reversely catalyse ATP production from G6P when the cytoplasmic ATP concentration reaches a certain threshold. Only ANT2 precisely allows ATP to be transported inwards into the mitochondrial matrix to maintain tumor cell survival and apoptotic resistance (52) (**Figure 4**). However, other ANTs, mainly located in nontumor cells, can continuously export mitochondrial ATP to the cytoplasm, which is detrimental to tumor cells with mitochondrial dysfunction. Given that the major ANT subtypes are different in tumor cells and nontumor cells, there may be discrepancies in the mechanisms that mediate CypD-ANT binding and the subsequent downstream effects.

### Inhibiting Tumor Cell Apoptosis

Inhibition of tumor cell apoptosis is an important prerequisite and marker of tumorigenesis. CypD overexpression has been reported to attenuate apoptosis. As early as 2004, Schubert A et al. found that CypD was significantly upregulated in various tumors of reproductive tissues (breast, uterus and ovary) and inhibited apoptosis of tumor cells (11). This study found that CypD may attenuate the activation of mPTP triggered by other proapoptotic stimuli and the release of pro-apoptotic factors in a manner independent of PPIase activity, thus promoting tumor cell survival. Unfortunately, the protective mechanism of CypD on tumor cells was not specifically elucidated in this study. The formation of mtHK II is known to activate metabolic pathways such as aerobic glycolysis and pentose phosphate pathways to produce sufficient ATP and metabolic intermediates (such as NADPH) to rapidly provide energy and biosynthetic substrates for tumor cell growth and invasion. Furthermore, mtHK II can competitively bind VDAC with the proapoptotic factor Bax to inhibit the translocation of Bax to mitochondria, thus antagonizing Bax-mediated apoptosis (53, 54). Intriguingly, Machida et al. found that mtHK II formation is necessary for CypD to inhibit apoptosis of HeLa cells (human cervical cancer cell line) and C6 cells (rat glioma cell line), while PPIase activity is essential for CypD to stabilize mtHK II (47). This is inconsistent with the results of Schubert et al., which may be attributed to different tissue sources of tumor cells or diverse upstream signalling pathways that regulate CypD. In addition, the binding of CypD and Bcl-2 enhances the limiting effect of Bcl-2 on cytochrome C (Cyto C) release and improves the antiapoptotic effect of various tumor cell lines (human osteosarcoma cell line Saos2 and human acute leukemia cell line HL60) (55). Overexpression or knockdown of CypD can upregulate or downregulate the resistance of tumor cells to apoptotic stimulation, respectively (56). Coincidentally, downregulated miRNA-27b-3p in oral mucosal basal cells of patients with oral lichen planus (a typical precancerous lesion) also enhances the interaction of CypD-Bcl2, inhibits the release of proapoptotic factors and accelerates deterioration (57).

As previously mentioned, the association of CypD-ANT2-VDAC1-mtHK II contributes to mPTP opening to ensure either continuous ATP output for mtHK II utilization or ATP input for the maintenance of intramitochondrial enzymatic pathways. How do tumor cells avoid mPTP-mediated cell apoptosis or necrosis while maintaining the essential aerobic glycolysis through the CypD-mPTP axis? We hypothesize that the degree of mPTP opening for maintaining ATP flow is confined, and that cytochrome C and apoptosis-inducing factor accompanying ATP output are not sufficient to initiate the caspase cascade-mediated mitochondrial apoptosis pathway. Indeed, although some degree of mitochondrial swelling and decreased MMP has been reported in LM7 and 143B osteosarcoma cells, cytochrome C levels in the cytoplasm do not exceed those in noncancerous hFOB cells (44). In addition, although mPTP opening can interfere with OXPHOS, tumor cells mainly rely on aerobic glycolysis to produce ATP. Aerobic glycolysis can fully improve the utilization rate of glucose, rapidly supply energy to tumor cells, and improve mitochondrial dysfunction by reversing the delivery of ATP to mitochondria. Thus, tumor cells maintain the essential MMP and ATP levels in a virtuous cycle to avoid cell necrosis induced by ATP depletion (**Figure 4**).

### Inducing Tumor Metastasis and Invasion

p53 is a typical tumor suppressor gene. Different forms of p53 mutations can affect the tumor suppressor or transcriptional activity of p53 and may even promote tumor progression. TP53 truncating mutations are common in human tumors, especially TP53 exon-6 truncating mutations. p53 exon-6 truncating mutants are similar to the naturally occurring selective p53 splice variant (p53-psi), which lacks transcriptional activity and responds to DNA damage. However, they can translocate into mitochondria, bind to CypD and activate mPTP opening (58). Traditionally, mPTP opening suppresses mitochondrial function and triggers apoptosis. Interestingly, however, the CypD-mPTP axis activated by p53 exon-6 truncating mutants or p53-psi could promote tumorigenesis and present malignant features such as downregulation of E-cadherin expression. Likewise, p53ψ derived from the use of alternative 3’ splice site in p53 intron 6 could also enhance the motility and invasive capacity of multiple lung cancer and breast cancer cell lines by activating the CypD-mPTP axis (59). Elevated ROS levels provoked by mPTP overopening played a pivotal role in p53ψ-induced epithelial-mesenchymal transformation (EMT).

Considering that the vast majority of p53 mutations that occur in human cancers are missense, the role of p53 missense mutants in regulating CypD is of interest to us. So far, only one article has mentioned both p53 missense mutants and CypD (60). It was found that mitochondrial wild-type p53 protein severely damaged the integrity of MOM and MIM by inducing the oligomerization of Bax, Bak and VDAC and the endogenous complex formation with CypD, respectively, thereby promoting the release of apoptotic factors. However, tumor-derived p53 missense mutants lost the ability to activate the Bax/Bak lipid pore. Unfortunately, the association between p53 missense mutants and CypD has not been further studied. Therefore, do missense mutations of p53 affect the association with CypD? If so, what are the downstream effects of this association? Are all or only some forms of p53 missense mutations associated with CypD? These seem to be very promising questions.

### Facilitating Tumor Resistance

The phosphatidylinositol-3 kinase (PI3K) pathway is a convergence point that regulates cell proliferation, survival and bioenergetics and is often used in tumor therapy. It has been reported that Akt2 abnormally activated by PI3K small molecule inhibitors (PI3Ki) in glioblastoma cells can translocate to mitochondria and subsequently phosphorylate CypD at S31 (61). Phosphorylated CypD supported mitochondrial bioenergetics, inhibited tumor cell apoptosis, and thereby mediated resistance to PI3K therapy. When combined with gamitrinib (a mitochondrial homeostasis inhibitor), PI3Ki effectively eliminated PI3K/Akt2/CypD pathway-mediated tumor resistance and significantly induced glioblastoma cell apoptosis.

## Negative Effect of CypD on Tumor Progression

**Figure 5:**
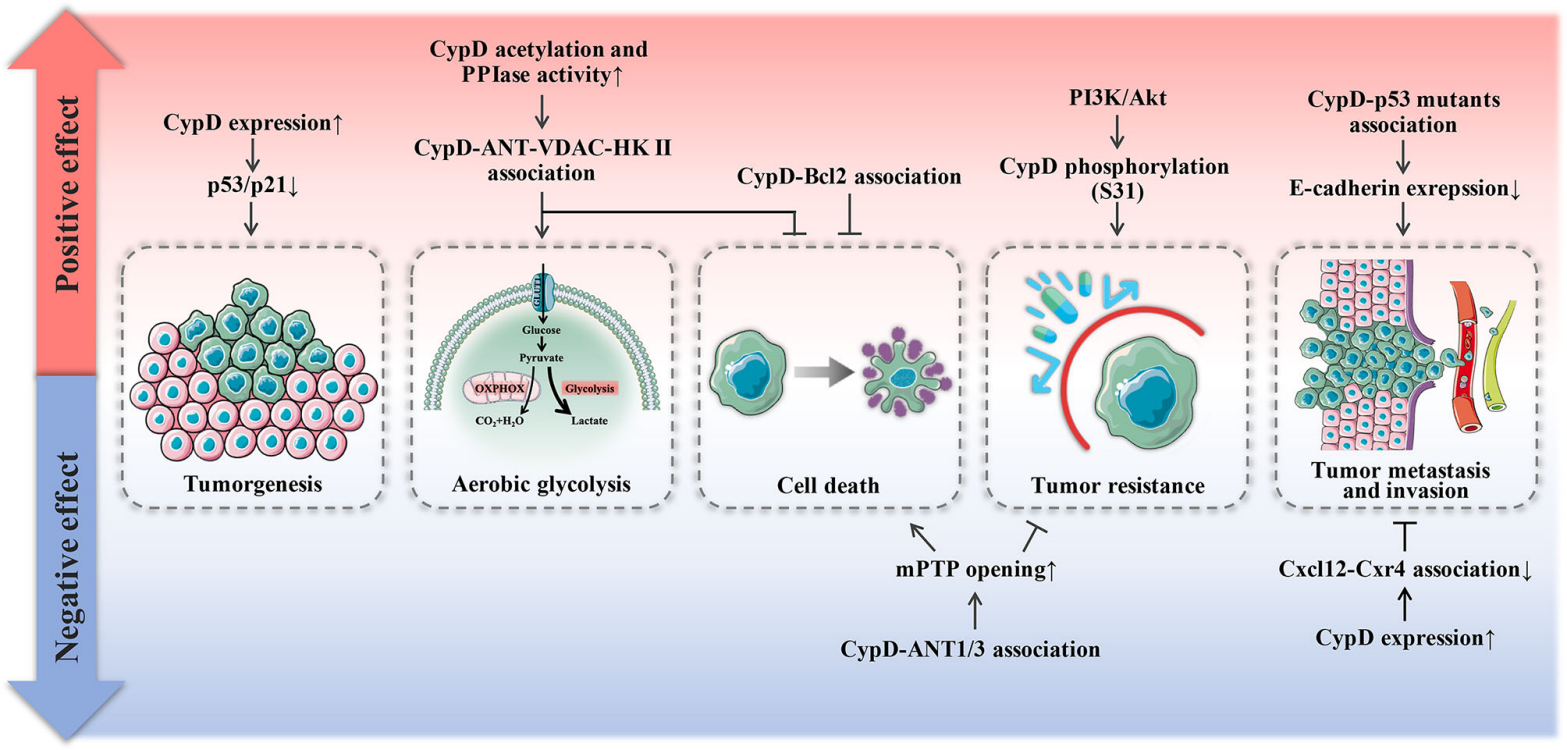
Dual effect of CypD on tumor progression. Red areas represent promotional mechanisms, and blue areas represent inhibitory mechanisms.

As shown in **Figure 5**, the role of CypD in tumor development is dual. Even for the same dimension of tumor development, CypD provoked by disparate upstream signals will exhibit bidirectional influence on tumors.

### Promotion of Tumor Cell Death

**Figure 6:**
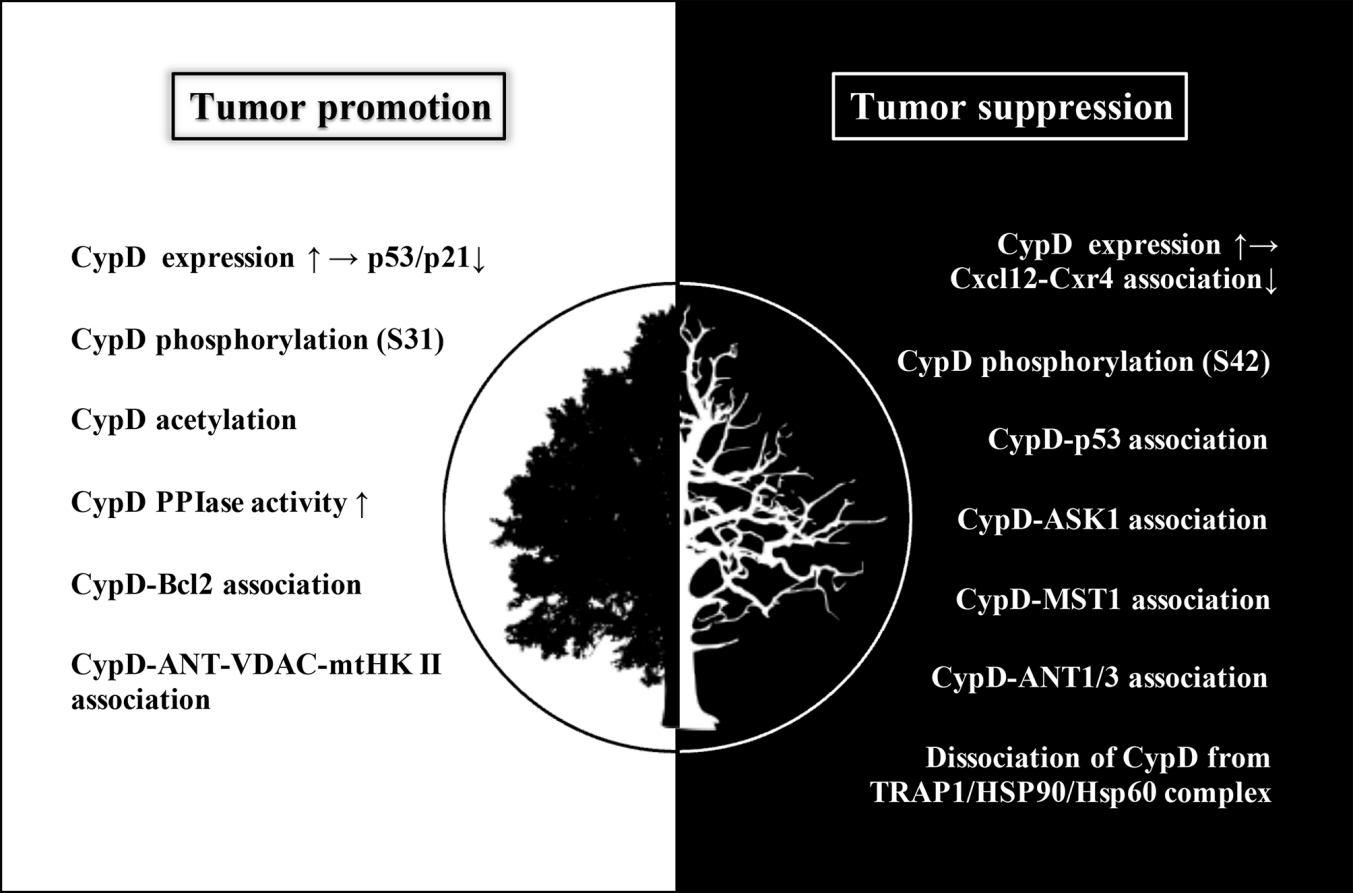
The white area (left) shows the modulations of CypD that promote tumor progression, mainly including inhibition of p53/p21 pathway mediated by upregulated CypD expression, CypD phosphorylation (S31), CypD acetylation, increased CypD PPIase activity, CypD-Bcl2 association and CypD-Ant-VDAC-mtHK II association. The black area (right) shows the modulations of CypD that suppress tumor progression, mainly including inhibition of Cxcl12-Cxr4 association mediated by upregulation of CypD expression, CypD phosphorylation (S42), association of CypD with several binding partners (p53, ASK1, MST1, ANT1/3), and dissociation of CypD from TRAP1/HSP90/HSP60 complex. Although increased CypD expression may occur in both tumor promotion and suppression, its downstream pathways are different, subsequently leading to two distinct cellular events. Elevated CypD expression actuates tumorigenesis by inhibiting p53/p21 pathway on the one hand, and hampers tumor metastasis and invasion by interfering Cxcl12-Cxr4 association on the other hand.

As shown in **Figure 6**, most antitumor drugs can induce tumor cell necrosis by activating the CypD-mPTP axis (62–70). For example, icaritin could induce necrosis of colon tumor cells, but not apoptosis, which is dependent on activation of the JNK pathway (71). It was found that CypD-ANT1 association enhanced mPTP opening, resulting in increased mitochondrial depolarization and the release of lactate dehydrogenase into the cytoplasm. Indeed, CypD-ANT1 association was also involved in the therapeutic mechanisms of several other antitumor drugs (72–74). Furthermore, both bishonokiol A and 1, 2-Diarachidonoyl-Sn-glycero-3-phosphoethanolamine (DAPE) upregulated CypD expression by activating the RIP1/RIP3/MLKL necrosis cascade, thus promoting mPTP-mediated necrosis of breast cancer and malignant pleural mesothelioma cells (75, 76). Similarly, bromocriptine could also promote the phosphorylation of CypD through the RIP3/MLKL pathway, thus inducing necrosis of prolactinoma cells (77). Moreover, CypD-p53 association has also been reported to be involved in the process of tumor cell necrosis induced by various antitumor drugs (78–83).

Moreover, CypD can also mediate the apoptosis process of tumor cells induced by antitumor drugs (84–93). For example, phosphorylated GSK3β is known to competitively bind ANT1 with CypD, thereby inhibiting mPTP opening. However, hirsutine dephosphorylated GSK3β in lung cancer cells through the ROCK1/PTEN/PISK/AKT pathway and activated the CypD-mPTP axis, thereby resulting in MMP decline, ATP dissipation, and caspase cascade-triggered apoptosis (94). Consistently, esculetin could activate the CypD-mPTP axis and induce apoptosis of gastric cancer cells by upregulating intracellular oxidative stress levels (69). In addition, tumor necrosis factor receptor-associated protein (TRAP1), a major member of the mitochondrial heat shock protein 90 (HSP90) family, can trap CypD in the TRAP1/HSP9/HSP60 multichaperone complex, thus limiting the translocation of CypD to the MIM. Treatment with HSP60 siRNA could promote the escape of CypD from the above complex, restore its activity and activate the excessive opening of mPTP, resulting in multiple tumor cell apoptosis (95). It should be noted that antitumor drugs do not induce just one type of cell death when treating a given tumor. For example, the enhanced association of p53-CypD-ANT1 led to both apoptosis and necrosis in non-small-cell lung cancer cells after treatment with ASP4132 (96). Inhibition or knockdown of CypD reversed cell necrosis induced by ASP4132 without affecting cell apoptosis. As mentioned above, the endogenous complex formed by wild-type p53 and CypD can participate in the destruction of MIM’s integrity. P53 is not only a typical tumor suppressor gene, but also regarded as a stress sensor that can sense multiple insults. For example, in response to oxidative stress during ischemia-reperfusion injury, p53 translocated into the mitochondrial matrix and triggered brain tissue necrosis by forming a robust complex with CypD (97).

In addition to the above two common forms of tumor cell death, CypD-induced autophagic cell death has been observed in liver cancer cells treated with andrographolide (98). This is because the activation of the CypD-mPTP axis by andrographolide leads to an increase in LC3 II and autophagosome in hepatoma carcinoma cells. Activation of CypD-mPTP axis is a common mechanism of tumor cell death induced by most antitumor drugs which is primarily achieved by upregulating the CypD-ANT1/3 association. As previously described, ANT1/3 can continuously export ATP into the cytoplasm, causing bioenergetic collapse of dysfunctional mitochondria in tumor cells and subsequently triggering cell death (99).

### Attenuating Tumor Metastasis and Invasion

In contrast, Tavecchio et al. found that CypD expression was reduced or even lost in a variety of tumor cell lines (human glioma cell line LN229, human breast cancer cell line MCF-7 and human pancreatic cancer cell line MiaPACA), which could obviously activate interorganelle signalling and the pleiotropic inflammatory mediator STAT3 (100). On the one hand, activated STAT3 enhanced tumor cell proliferation by accelerating entry into S-phase. On the other hand, the Cxcl12-Cxcr4 association mediated chemokine-dependent autocrine/paracrine cell motility after CypD ablation, which was manifested as enhanced tumor cell migration and invasion. This finding indicates that CypD can inhibit tumor metastasis to some extent.

### Suppressing Tumor Resistance

Wu et al. found that mortalin reduced mitochondrial permeability by blocking CypD-ANT3 binding and promoted the proliferation of human B-Raf^V600E^ melanoma cells and their resistance to vemurafenib (101, 102). Moreover, CypD expression and its association with mammalian sterile 20-like kinase 1 (MST1) were significantly downregulated, mediating gemcitabine resistance in pancreatic tumor cell lines (103). These data suggest that the association of CypD with its binding partners may be involved in the chemoresistance of some tumors.

## Conclusions and Perspectives

CypD is recognized as a gate that regulates cell fate and energy metabolism by many mechanisms. It has been demonstrated that CypD can affect tumor progression in multiple ways and seems to play a dual role in tumor cell fate (**Table 1** and **2**). CypD-mediated transient opening of mPTP helps regulate calcium homeostasis and attenuate ROS accumulation in mitochondria to maintain mitochondrial activity. Moderate levels of mPTP opening continuously provide substrates for aerobic glycolysis and essential intramitochondrial enzymatic pathways in tumor cells. The modestly activated CypD-mPTP axis can also promote the survival, metastasis and invasion of tumor cells. However, excessive mPTP opening inhibits the bioenergetics and malignant characteristics of tumor cells and triggers various forms of tumor cell death. This regulatory feature of the CypD-mPTP axis has been used to develop a variety of antitumor drugs. Therefore, the complex regulatory mechanisms of CypD make it a promoter of tumor progression, but also a weapon by which antitumour drugs may kill tumor cells (**Figure 6**). Rational utilization of the biological functions of CypD may make CypD a hot target for the treatment of various diseases, including tumors, in the future.

**Table 1:** Positive effect of CypD on tumor progression.

Dimension	Upstream signal	Modulation of CypD	Outcome	Tumor type	Reference
Tumorigenesis	Ras/Raf-1/MEK/ERK pathway	CypD expression↑	Promotion of tumorigenesis and cell growth	Lung cancer, Breast cancer	(37)
Metabolic reprogramming	SIRT3↓	CypD acetylation, PPIase activity↑, CypD-ANT2-VDAC-mtHK II association	Promotion of aerobic glycolysis and maintenance of the essential intramitochondrial enzymatic pathways	Breast cancer, Colon cancer	(45, 46)
Tumor cell death	Unmentioned	CypD expression↑	Inhibition of pro-apoptotic factors release	Tumors of reproductive tissues, Meningioma	(11, 56)
	SIRT3↓	CypD acetylation, PPIase activity↑, CypD-ANT2-VDAC-mtHK II association	Inhibition of apoptosis	Colon cancer	(46)
	Unmentioned	PPIase activity↑	Inhibition of Bax-induced apoptosis	Breast cancer, Glioma	(47)
	Unmentioned	CypD-Bcl2 association	Inhibition of cytochrome C release	Osteosarcoma, Leukemia	(55)
	PI3Ki/Akt2	CypD phosphorylation (S31)	Inhibition of tumor cell apoptosis	Glioblastoma	(61)
Tumor metastasis and invasion	p53 mutations	CypD-p53 mutations association	Promotion of motility and invasive capacity of tumor cells	Lung cancer, Breast cancer, Melanoma	(58, 59)
Tumor resistance	PI3Ki/Akt2	CypD phosphorylation (S31)	Promotion of resistance to PI3K therapy	Glioblastoma	(61)

**Table 2:** Negative effect of CypD on tumor progression.

Dimension	Antitumor drug	Upstream signal	Modulation of CypD	Outcome	Tumor type	Reference
Tumor cell death	Hirsutine	ROCK1/PTEN /PI3K/GSK3β	CypD-ANT1 association	Induction of apoptosis	Lung cancer	(94)
	GSK1059615	PI3K/Akt/mTOR↓	CypD-ANT1 association	Induction of necrosis	Head and neck squamous cell carcinoma	(72)
	ASP4132, AICAR	AMPK	CypD-ANT1 association	Induction of necrosis	Non-small cell lung cancer, Prostate cancer	(73, 96)
	Icaritin	JNK	CypD-ANT1 association	Induction of necrosis	Colorectal cancer	(71)
	Mortalin deleption	MEK/ERK	CypD-ANT1 association	Induction of cell death	B-Raf^V600E^ melanoma, KRAS tumor	(74, 101)
	Resminostat	mTOR↓	CypD-ANT1 association	Induction of apoptosis	Hepatocellular carcinoma	(84, 85)
	NPC-26, ABT-737, Curcumin	Unmentioned	CypD-ANT1 association	Induction of apoptosis	Pancreatic cancer, Melanoma	(86–88)
	DN3	Unmentioned	CypD expression↑	Induction of apoptosis and cell cycle arrest	Gastric cancer	(89)
	Sorafenib	p-ERK↓	CypD expression↑	Induction of apoptosis	Clear cell-renal cell carcinoma	(90)
	Bishonokiol A	RIP1/RIP3/MLKL	CypD expression↑	Induction of necrosis	Breast cancer	(75)
	Bromocriptine	RIP1/RIP3/MLKL	CypD phosphorylation	Induction of cell death	Prolactinoma	(77)
	DAPE	RIP1	Unmentioned	Induction of necrosis	Malignant pleural mesothelioma	(76)
	Emodin	ERK	CypD-emodin association	Induction of apoptosis	Hepatocellular carcinoma	(91)
	C6+Docetaxel	AMPK, JNK, HER/ERK/Akt	Unmentioned	Inhibition of cell growth and induction of apoptosis	Breast cancer	(92)
	Berberine, Doxorubicin, Salinomycin, Cisplatin, PF-543	p53	CypD-p53 association	Induction of necrosis	Prostate cancer, Lung cancer, Glioma, Pancreatic cancer, Colorectal cancer	(78–83)
	Gemcitabine	MST1	CypD-MST1 association	Induction of necrosis	Pancreatic cancer	(103)
	4SC-202	ASK1	CypD-ASK1 association	Induction of apoptosis	Hepatocellular carcinoma	(93)
	Hsp60 RNAi	Unmentioned	Dissociation of CypD from TRAP1-HSP90-HSP60 complex	Inhibition of cell growth and induction of apoptosis	Breast cancer, Colorectal cancer, Glioblastoma	(95)
Tumor metastasis and invasion	none	Unmentioned	CypD expression	Inhibition of cell proliferation and invasion	Glioblastoma, Breast cancer	(100)
Tumor resistance	Mortalin deleption	MEK/ERK	CypD-ANT1 association	Antagonism of resistance to vemurafenib	B-Raf^V600E^ melanoma	(101, 102)
	Gemcitabine	MST1	CypD-MST1 association	Antagonism of resistance to gemcitabine	Pancreatic cancer	(103)

## Author Contributions

LZ is responsible for drafting of the paper and drawing the figures. BZ is responsible for the conception and review of the paper. YL, RZ, BH and WW are responsible for the writing, review and revision of the manuscript. All authors contributed to the article and approved the submitted version.

## Funding

This study was supported by grants from the National Natural Science Foundation of China (No. 82173371), Tai Shan Young Scholar Foundation of Shandong Province (No.tsqn201909192) and Natural Science Foundation of Shandong Province (No. ZR2021QH294).

## Conflict of Interest

The authors declare that the research was conducted in the absence of any commercial or financial relationships that could be construed as a potential conflict of interest.

## Publisher’s Note

All claims expressed in this article are solely those of the authors and do not necessarily represent those of their affiliated organizations, or those of the publisher, the editors and the reviewers. Any product that may be evaluated in this article, or claim that may be made by its manufacturer, is not guaranteed or endorsed by the publisher.
